# The Complete Mitochondrial Genome of Deep-Sea Snipe Eel *Nemichthys curvirostris* (Anguilliformes: Nemichthyidae)

**DOI:** 10.3390/genes16121498

**Published:** 2025-12-15

**Authors:** Xin Jin, Yanqing Ma, Lingzhi Li, Zhiwei Yuan, Chunyan Ma, Fengying Zhang, Wei Chen, Hanfeng Zheng, Chao Li, Zhi Zhu, Ming Zhao

**Affiliations:** 1 Key Laboratory of East China Sea Fishery Resources Exploitation, Ministry of Agriculture, East China Sea Fisheries Research Institute, Chinese Academy of Fishery Sciences, 300 Jungong Road, Shanghai 200090, China; jx18036195921@163.com (X.J.); mayanqing618@126.com (Y.M.); lilz@ecsf.ac.cn (L.L.); macy@ecsf.ac.cn (C.M.); zhangfy@ecsf.ac.cn (F.Z.); chenw@ecsf.ac.cn (W.C.); zhenghf@ecsf.ac.cn (H.Z.); lic@ecsf.ac.cn (C.L.); zhuz@ecsf.ac.cn (Z.Z.); 2 Weihai Customs District, Weihai Customs of the People’s Republic of China, No. 64 Haibin North Road, Huancui District, Weihai 264200, China; yzuyzw@126.com

**Keywords:** *Nemichthys curvirostris*, nemichthyidae, mitochondrial genome, phylogeny

## Abstract

**Background:** Snipe eels (family Nemichthyidae) are a group of pelagic fishes with unique specializations; yet, species within this study are not well-studied due to a lack of molecular data. As typical mesopelagic-to-bathypelagic fishes, snipe eels exhibit extreme body elongation, reduced skeletal ossification, and highly specialized beak-like jaws that facilitate survival in deep-sea midwater environments. **Methods:** The complete mitochondrial genome of the deep-sea eel *Nemichthys curvirostris* (Anguilliformes: Nemichthyidae) was sequenced and annotated, representing the first mitogenomic resource for this species. The phylogenetic position of *N. curvirostris* was also explored. **Results:** The circular genome of *N. curvirostris* was determined to be 16,911 bp in length and contained 37 genes, including 13 protein-coding genes, 22 tRNAs, 2 rRNAs, and a single control region, with an overall A + T bias of 56.67%. The maximum-likelihood phylogeny inferred from concatenated mitochondrial protein-coding genes recovered a well-supported monophyletic *Nemichthys* clade, with *N. curvirostris* positioned as the sister taxon to *N. scolopaceus*. The genera *Avocettina* and *Labichthys* were recovered as sister taxa, and Nemichthys clustered within a broader clade alongside them. The COX1 haplotype phylogeny showed that the two public database sequences (HQ563894.1 and MN123435.1) appeared as long, isolated branches outside the main *N. curvirostris* lineage, with COX1 genetic distances from typical *N. curvirostris* haplotypes reaching 12–13%, far exceeding the expected range of intraspecific variation. **Conclusions:** This mitogenome provides a valuable molecular resource for phylogenetic, evolutionary, and population genetic studies of deep-sea Anguilliformes.

## 1. Introduction

The family Nemichthyidae, commonly known as snipe eels, comprises a group of highly specialized pelagic fishes adapted to life in the mesopelagic and bathypelagic zones of tropical and temperate oceans [1]. These eels exhibit a suite of remarkable morphological specializations, including extremely elongated, scaleless bodies, exceptionally high vertebral counts, and non-occluding, beak-like jaws [2]. This type of jaw is a feature present in all individuals except fully mature males, in which the jaws become occlusible [2,3]. Currently, three genera (*Avocettina*, *Labichthys*, and *Nemichthys*) and nine valid species are recognized within Nemichthyidae [4]. Despite their unique adaptations and broad oceanic distribution, members of this family remain relatively understudied, particularly at the molecular level.

Among snipe eels, the boxer snipe eel *N. curvirostris* [5] is notable for its wide distribution across several major ocean basins, including the Indian Ocean. Despite being reported in multiple regional surveys [6,7], comprehensive molecular data for *N. curvirostris*, as well as most members of Nemichthyidae, remain scarce. Within Anguilliformes, Nemichthyidae has historically been placed as the sister family to Serrivomeridae, a group of deep-sea eels characterized by needle-like jaws, reduced dentition, and extreme morphological streamlining [8]. Morphological studies have consistently recovered *Nemichthys* as closely aligned with serrivomerid taxa based on shared cranial and vertebral modifications, leptocephalus morphotypes, and reductions in branchial and suspensorial elements [9]. Independent molecular analyses using COX1, 12S, 16S, and several nuclear markers have also supported this affinity, placing *Nemichthys* within a well-resolved clade alongside *Avocettina*, *Labichthys*, and Serrivomeridae [10]. Beyond that, the current understanding of this family is primarily based on morphological observations. The difficulties in transporting samples of deep-sea organisms have further limited the availability of genomic resources, including complete mitochondrial genomes, which are essential for reconstructing phylogenetic relationships within the family. Due to the maternal inheritance features of mitochondrial genomes, they provide a practical framework for generating species-specific genetic markers and establishing a foundation for taxonomic studies [11].

In light of the scarce molecular studies of Nemichthyidae, we sequenced, assembled, and annotated the complete mitochondrial genome of *N. curvirostris* from a specimen collected in the Northeast Indian Ocean. An Anguilliformes phylogenetic tree was also constructed to elucidate the phylogenetic position of *N. curvirostris*. This study provides the first full mitochondrial reference for the species, which is a valuable resource for advancing taxonomic resolution, phylogenetic inference, and evolutionary investigations within Nemichthyidae and related deep-sea lineages.

## 2. Materials and Methods

### 2.1. Sample Collection and Morphological Identification

**Figure 1:**
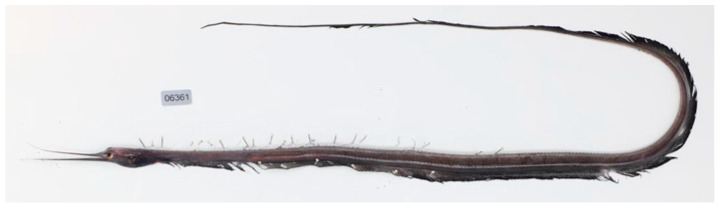
Habitus photograph of *N. scurvirostris* (Specimen No. 06361).

The specimen of *N. curvirostris* [5] was collected from the Northeast Indian Ocean (3.2982°N, 66.2986°E) during the three-month 2024 Northwest Indian Ocean scientific expedition aboard R/V Lanhai 201 (Figure 1). Immediately following capture, a portion of muscle tissue was excised and preserved in absolute ethanol to ensure the quality of DNA for downstream analyses. The entire specimen (catalog number: 06361) was subsequently fixed and stored in the East China Sea Fisheries Research Institute, Chinese Academy of Fishery Science, Shanghai, China, under catalog number: 06361. Species identification was conducted following established taxonomic keys and descriptions [1,2,3]. Diagnostic features of *N. curvirostris* include a markedly elongated, scaleless body, distinctive curvature of the beak-like jaws, the relative positioning and confluent nature of the dorsal, anal, and caudal fins, and the presence of pectoral fins.

### 2.2. Genomic DNA Extraction and Sequencing

Genomic DNA was isolated from ethanol-preserved muscle tissue using the Fish DNA extraction kit (Qingdao Insight Bio Co., Ltd., Qingdao, China). DNA quality and concentration were evaluated using a NanoDrop 2000 spectrophotometer (Thermo Scientific, Waltham, MA, USA), and integrity was verified via agarose gel electrophoresis. Paired-end sequencing libraries, targeting an insert size of ~350 bp, were constructed using the TruSeq DNA PCR-Free Library Prep Kit (Illumina, San Diego, CA, USA) according to the manufacturer’s protocol. Library quality was assessed using the Agilent Bioanalyzer (Agilent, Santa Clara, CA, USA) and library quantification was performed via qPCR. Sequencing was conducted on the Illumina HiSeq X Ten platform, generating 150 bp paired-end reads. The initial sequencing generated 7.5 Gb of raw data, of which 7 Gb were retained following quality filtering. Adapter removal and quality control were performed using Fastp v0.20.0 [12], resulting in 5.5 Gb high-quality paired-end reads, which were subsequently used for mitochondrial genome assembly. For mitochondrial genome reconstruction, approximately 10% of the total reads were subsampled from the nuclear genome sequencing dataset.

### 2.3. Mitogenome Assembly and Annotation

De novo assembly of the mitochondrial genome was performed using MITObim v1.9.1 [13]. Annotation included identification of all 13 protein-coding genes (PCGs), 22 tRNA genes, two rRNA genes, and the control region (D-loop), based on sequence homology and the vertebrate mitochondrial code. The assembled mitochondrial genome was annotated through a rigorous multi-validation pipeline. Initial gene predictions were performed using MitoAnnotator [14] on the MITOS Web Server [15], followed by cross-verification against homologous sequences from closely related taxa via BLAST v2.14.0 searches. Protein-coding genes (PCGs) were carefully examined to confirm accurate start and stop codon positions under the vertebrate mitochondrial genetic code, and translations were checked to ensure the absence of internal stop codons. Transfer RNA (tRNA) genes were further validated using tRNAscan-SE v2.0 [16], and ribosomal RNA genes were identified based on sequence homology and secondary structure predictions.

Key genomic features, including total genome length, base composition, gene order, and strand distribution, were summarized and compared with canonical vertebrate mitochondrial genomes. The nucleotide composition and relative synonymous codon usage (RSCU) of the 13 PCGs were assessed with MEGA 12 [17]. Nucleotide compositional skew was calculated as AT skew = [(A − T)/(A + T)] and GC skew = [(G − C)/(G + C)] [18].

### 2.4. Phylogenetic Analyses

To determine the evolutionary relationships of *N. curvirostris*, maximum-likelihood (ML) phylogenetic reconstruction was conducted using a concatenated dataset of the 13 mitochondrial protein-coding genes (excluding *ND6*). All taxa included in the phylogenetic reconstruction are represented by complete mitochondrial genomes. In addition, the genome data of species in all families of anguilliform and genus *Nemichthys* were used if their mitochondrial genomes had been published. Individual PCGs were aligned with MAFFT v7.304 [19], and poorly aligned or ambiguously aligned regions were masked to reduce potential artifacts. ModelFinder identified the best-fitting nucleotide model as GTR + G + I [20]. ML analysis was carried out in IQ-TREE v2.1.2 [21]. Node support was evaluated with 1000 bootstrap replicates [22]. The resulting phylogenetic tree was visualized and annotated using the Interactive Tree of Life [23].

In addition to the mitogenome-based phylogeny, COX1 sequences of *N. curvirostris* were assembled to assess intraspecific haplotype structure. All COX1 sequences were retrieved from GenBank and aligned using MAFFT v7.304 [19] under the L-INS-i algorithm. Ambiguously aligned regions were masked, and the best-fit nucleotide model was selected using ModelFinder. ML reconstruction was performed in IQ-TREE v2.1.2 [21], and the resulting haplotype tree was visualized in the Interactive Tree of Life v7.0 [23]. Metadata including geographic origin and sequence quality were examined to interpret the placement of questioned COX1 entries.

## 3. Results

### 3.1. General Features of the N. curvirostris Mitochondrial Genome

**Figure 2:**
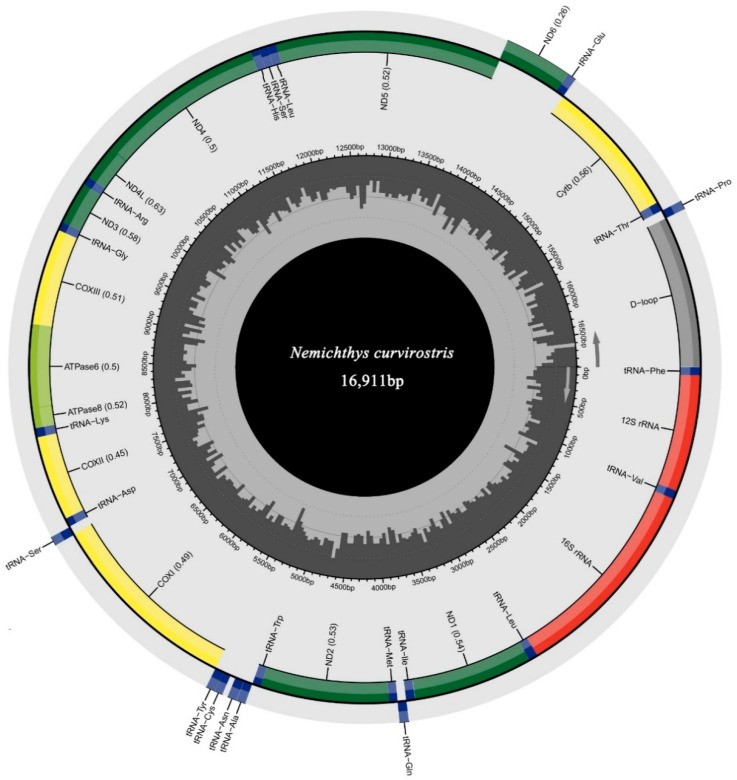
Circular map of the mitogenome of *N. curvirostris*. The tRNA genes are annotated based on their corresponding amino acid codes. The orientation of gene transcription is shown by arrows.

The complete mitochondrial genome of *N. curvirostris* (GenBank accession: PX571992) exhibited the typical circular, double-stranded structure with 16,911 bp in length (Figure 2). The mitogenome contained the standard complement of 37 genes, including 13 protein-coding genes (PCGs), 22 transfer RNA (tRNA) genes, two ribosomal RNA (rRNA) genes, and one non-coding control region (CR). Most genes, including all PCGs except *NAD6*, were encoded on the heavy (H) strand, while NAD6 and eight tRNA genes (*tRNA-Glu*, *tRNA-Pro*, *tRNA-Gln*, *tRNA-Ala*, *tRNA-Asn*, *tRNA-Cys*, *tRNA-Tyr*, and *tRNA-Ser*) were located on the light (L) strand. Nine gene overlaps were detected, ranging from 1 to 10 bp, with the largest located between *ATP8* and *ATP6*.

The nucleotide composition showed an apparent AT bias of 56.67%, with proportions of A  =  29.40%, T  =  27.27%, C  =  24.65%, and G  =  18.67% (Table 1). The corresponding AT-skew (0.038) and GC-skew (–0.138) values indicate a mild asymmetry of nucleotide composition.

### 3.2. PCGs and Codon Usage

The 13 PCGs spanned 11,339 bp of the genome, with *ND5* being the longest gene (1837 bp) and *ATP8* being the shortest (169 bp). Among the PCGs, twelve were initiated with the conventional start codon ATG, whereas *COX1* employed GTG as an alternative initiation codon. Stop codon usage was heterogeneous. Six genes (*NAD1*, *NAD4L*, *NAD6*, *ATP6*, *ATP8*, and *CYTB*) were terminated with the complete TAA codon, while four genes (*NAD2*, *NAD3*, *NAD5*, and *COX3*) were terminated with the standard TAG stop codon. *NAD4* and *COX2* were terminated with the incomplete codon T-- (Table 2).

**Figure 3:**
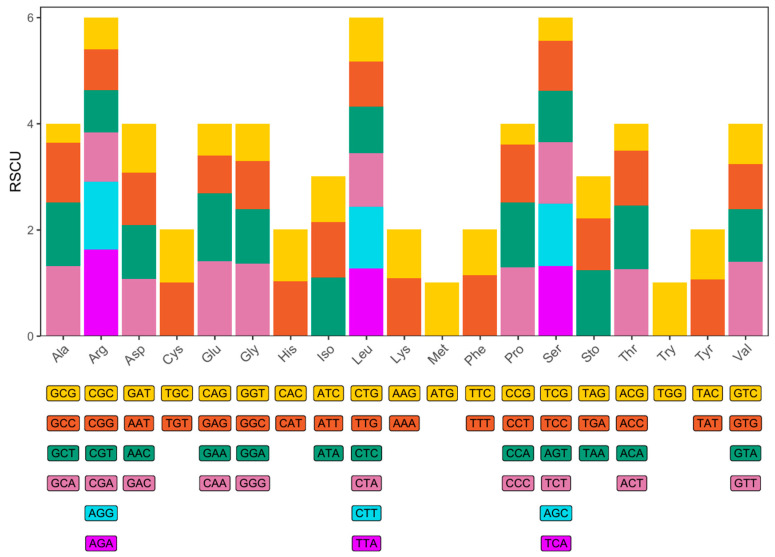
Relative synonymous codon usage (RSCU) of the *N. curvirostris* mitogenome. The *x*-axis shows the amino acids encoded and the codons that encode each amino acid. The *y*-axis shows the relative usage of synonymous codons.

A total of 5637 codons were identified, with Arg, Leu, and Ser being the most abundant amino acids (Figure 3). CAA, GTT, GGG, TAA, GAA, and TGA were the most frequently used codons, suggesting a preference for A and G over T and C. Codons ending in A were clearly overrepresented.

### 3.3. Transfer and Ribosomal RNA Genes

**Figure 4:**
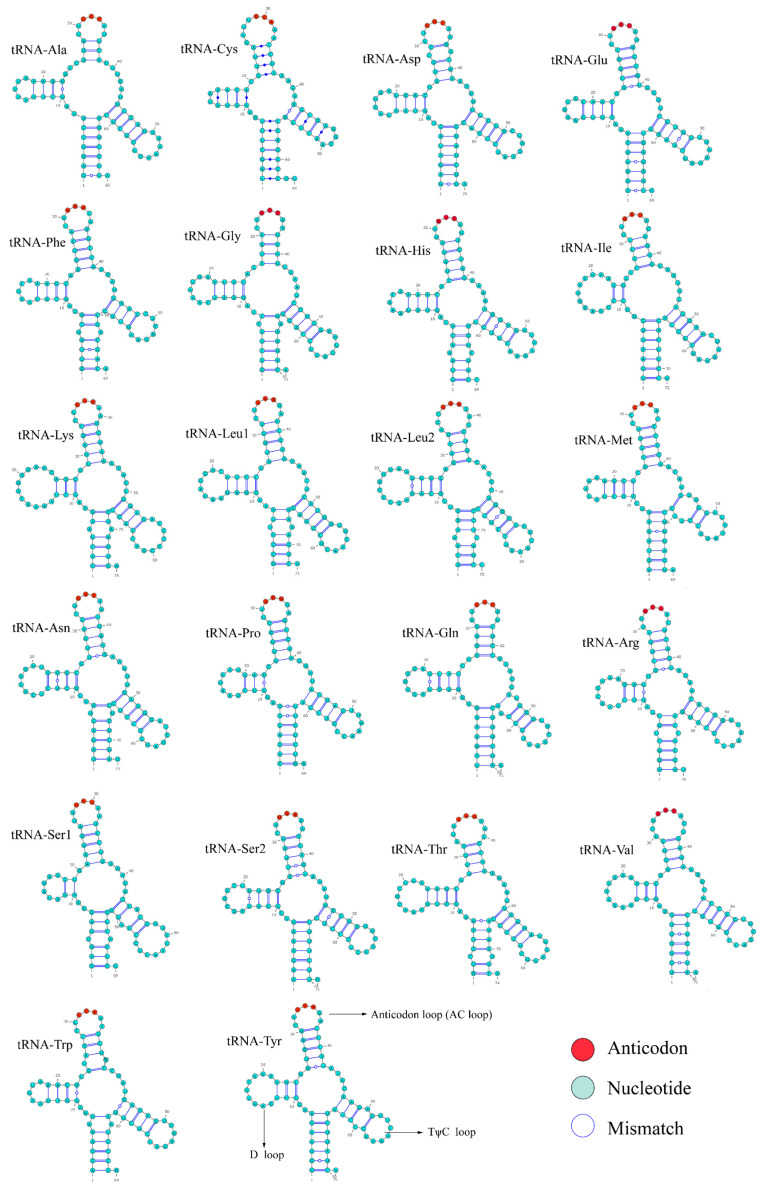
The predicted secondary structures of 22 tRNA genes in the *N. curvirostris* mitochondrial genome.

A total of 22 tRNA genes were identified and their typical secondary structures were successfully predicted in the mitochondrial genome (Figure 4). Among the 22 tRNAs, most possessed complete D and TΨC arms, whereas tRNA-Ser1 and tRNA-Ser2 exhibited truncated or incomplete arms, a simplified structural form commonly observed in vertebrate mitochondrial tRNAs. Base mismatches were mainly located at a few positions within the stem regions, but their low frequency did not affect the overall stability of the secondary structures. The 22 tRNA genes ranged from 63 to 75 bp with 1550 bp in total, constituting approximately 9.17% of the whole genome. Among them, *tRNA-CYS* was the shortest tRNA, while *tRNA-Lys* was the longest. Both AT and GC skews of the tRNA genes exhibited a positive value (Table 1). The two rRNA genes, *12S* and *16S rRNA*, were 950 bp and 1686 bp in length, respectively. They were situated between *tRNA-Phe* and *tRNA-Leu*, separated by *tRNA-Val*. Both genes showed a positive AT skew and negative GC skew (Table 1).

### 3.4. Control Region

The control region (CR), positioned between *tRNA-Pro* and *tRNA-Phe*, was characterized by a high A  +  T content (63.7%). It contained conserved sequence motifs, including CSB-F, CSB-D, and CSBs 1–3, which were typical of teleost mitochondrial control regions (Table 2).

### 3.5. Phylogenetic Analysis

**Figure 5:**
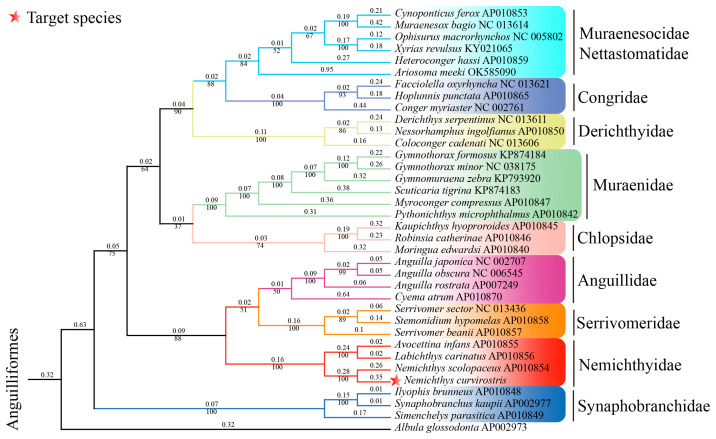
Maximum-likelihood (ML) phylogenetic tree of *N. curvirostris* and 34 other anguilliform species. Albula glossodonta represents the outgroup. Major anguilliform clades shown include Muraenidae, Congridae, Serrivomeridae, Nemichthyidae, Synaphobranchidae, and Ophichthidae, each recovered as monophyletic. Bootstrap support values are shown below nodes, and pairwise genetic distances are shown above nodes.

**Figure 6:**
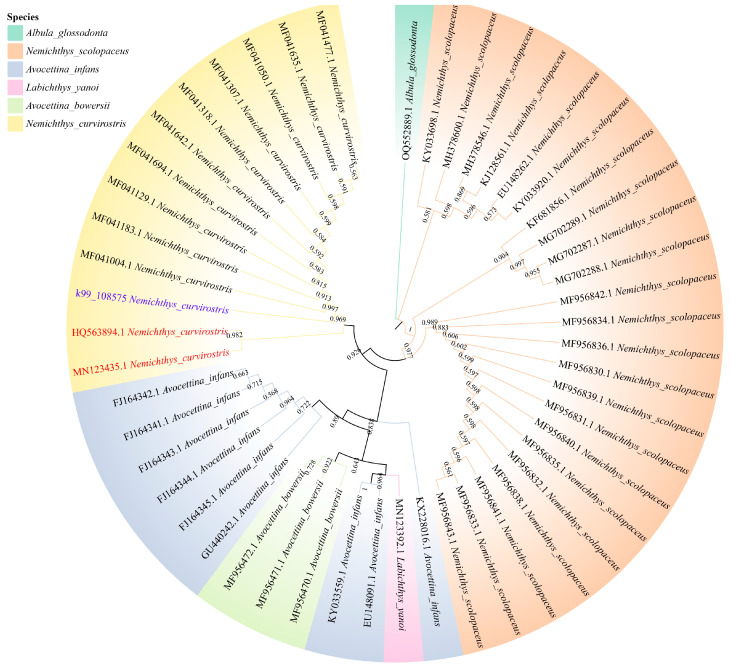
Maximum-likelihood (ML) haplotype phylogeny of *N. curvirostris* based on COX1 sequences. The questioned sequences are highlighted in red and the sequences generated in this study are shown in purple. Bootstrap support values are shown below nodes, and pairwise genetic distances are shown above nodes.

Phylogenetic analyses based on concatenated nucleotide sequences of the 13 PCGs (excluding *Nad6*) using the Maximum Likelihood method recovered a well-resolved topology (Figure 5). The family Nemichthyidae was retrieved as a strongly supported monophyletic clade (bootstrap  =  100%), forming a sister relationship with Serrivomeridae. Within Nemichthyidae, *Nemichthys* constituted a monophyletic genus, with *N. curvirostris* exhibiting the closest matrilineal affinity with *N. scolopaceus*, while *Avocettina* and *Labichthys* were recovered as sister taxa. To further complement the phylogenetic reconstruction based on the 12 mitochondrial protein-coding genes (PCGs), we additionally reconstructed a COX1 haplotype phylogeny. The newly generated COX1 sequence (k99_108575) clustered with the authenticated *N. curvirostris* reference sequences—excluding HQ563894.1 and MN123435.1—forming a well-supported monophyletic group (Figure 6). This sequence was fully consistent with the main *N. curvirostris* haplogroup, exhibiting a stable phylogenetic position with no indication of anomalous placement or deep lineage divergence. In contrast, the two public database records, HQ563894.1 and MN123435.1, failed to cluster within the primary *N. curvirostris* clade. Instead, each occupied a separate, distantly positioned side branch and did not form a supported lineage with one another. Pairwise K2P genetic distance analysis further showed that both sequences differed from typical *N. curvirostris* haplotypes by approximately 12–13%, far exceeding the expected range of intraspecific variation.

## 4. Discussion

This study provides a complete mitochondrial genome of *N. curvirostris*, which is the first comprehensive molecular resource for this widely distributed deep-sea eel. Its circular, double-stranded DNA of 16,911 bp, with a typical vertebrate gene content and structure, falls within the expected size range for teleost mitogenomes. The positive AT skew and negative GC skew of the whole genome suggest that adenine and cytosine are more abundant than thymine and guanine. These mild nucleotide skews are consistent with patterns observed in other eel species [24,25]. The conserved genome organization agrees with the general pattern observed in most Anguilliformes, although several eel families (e.g., Muraenesocidae, Congridae) have been reported to display varying degrees of mitochondrial gene rearrangement [26,27]. For codon usage, *N. curvirostris* exhibited a preference for codons terminating with the A nucleotide, and this pattern has been found in other Anguilliformes as well [26]. The use of GTG as an alternative start codon in *COX1* and the presence of incomplete stop codons (T--) in *COX2* and *NAD4* are consistent with the post-transcriptional polyadenylation mechanism commonly observed in teleost mitochondrial genes, whereby truncated stop codons are completed through the addition of a poly(A) tail during mRNA processing [28].

The mitochondrial genome structure of *N. curvirostris* exhibited a canonical gene composition and structural organization typical of teleost fishes. Generally, a teleostean group possesses unique or highly similar gene arrangements. Nevertheless, several types of gene arrangements have been detected in the mitogenomes of Anguilliformes [24,29]. Previous studies have reported mitochondrial gene rearrangements in species inhabiting specialized environments [30]. It has been suggested that random genomic rearrangements are generally deleterious and thus unlikely to contribute to structural stability [31]. However, the mitochondrial genome of *N. curvirostris* in this study retains the typical teleostean gene order, showing no evidence of gene rearrangement. Such genomic stability in *N. curvirostris* may reflect a relatively conservative mitochondrial evolution within Nemichthyidae.

Phylogenetic analysis based on concatenated sequences of the 13 protein-coding genes produced a well-supported topology that strongly confirmed the monophyly of Nemichthyidae, with Serrivomeridae as its sister group. Within Nemichthyidae, *Nemichthys* formed a distinct monophyletic genus, consistent with its morphological differentiation and wider distribution across multiple ocean basins. This pattern indicates a moderate level of divergence within Nemichthyidae, rather than an early split, and may reflect historical dispersal and ecological specialization. As a pelagic deep-sea eel with an exceptionally elongated body and specialized jaw morphology, *N. curvirostris* is well-adapted to life in midwater and bathyal zones. Such adaptations likely impose distinct metabolic demands related to hypoxia tolerance and energy efficiency. Mitochondria are important organelles for energy production [32], and their genes encode essential subunits of oxidative phosphorylation, making them particularly informative for understanding these physiological adaptations. Although no clear signal of gene rearrangement or accelerated evolution was detected in this study, further comparative genomic analyses across Nemichthyidae may reveal lineage-specific modifications associated with deep-sea environmental pressures. The COX1 haplotype phylogeny revealed that the two public database sequences (HQ563894.1 and MN123435.1) showed markedly aberrant patterns in our phylogenetic analysis. First, neither clustered with the validated *N. curvirostris* sequences but instead appeared on long, isolated terminal branches distant from the main clade. Second, their sequence similarity to typical *N. curvirostris* haplotypes was only around 80%, substantially lower than expected for conspecific sequences. Third, the COX1 genetic distances reached 12–13%, far exceeding the intraspecific variation commonly observed in deep-sea anguilliform fishes, and instead falling within levels typical of interspecific or even intergeneric divergence [33]. Moreover, the two anomalous sequences also differed considerably from each other (~6%) and did not form a coherent, well-supported lineage, failing to meet the monophyletic requirement expected for cryptic species. Given the substantial divergence of the two anomalous sequences, whether they represent cryptic species, subspecies, or other unrecognized lineages remains uncertain, and future studies with broader sampling and additional genetic markers will be essential to resolve their true taxonomic status.

## 5. Conclusions

In conclusion, the complete mitochondrial genome of *N. curvirostris* provides an important addition to the molecular data available for Nemichthyidae. Phylogenetic analyses support the monophyly of the family and confirm its placement as the sister group to Serrivomeridae within Anguilliformes. The mitogenome exhibits a conserved gene order and compositional pattern typical of teleosts, indicating structural and functional stability throughout evolution. This work offers a robust genomic framework for future investigations into the evolutionary history, systematics, and ecological adaptations of snipe eels and other deep-sea anguilliform taxa.

## Figures and Tables

**Table 1:** Nucleotide composition and AT and GC skews of the mitogenome of *N. curvirostris*.

Region	Length (bp)	A%	T%	C%	G%	(A + T)%	(G + C)%	AT Skew	GC Skew
Genome	16,911	29.4	27.27	24.65	18.67	56.67	43.32	0.038	−0.138
PCGs	11,326	27.05	29.77	24.67	18.51	56.82	43.18	−0.048	−0.143
tRNA	1550	27.74	27.10	20.90	24.26	54.84	45.16	0.012	0.074
rRNA	2635	33.85	20.30	23.61	22.24	54.15	45.85	0.250	−0.030
D-loop	1400	31.23%	32.44%	20.55%	15.78%	63.67%	36.33%	−0.019	−0.134

**Table 2:** Characteristics of the mitochondrial genome of *N. curvirostris*. “+” and “−” represent genes located on the forward and reverse strands of the mitochondrial genome, respectively.

Gene	Nucleotide Positions	Size (bp)	Strand	Intergenic Nucleotides	Start	Stop
COX3	140–926	787	+	−2	ATG	TAG
tRNA^Gly^	925–996	72	+	−1		
NAD3	996–1347	352	+	−3	ATG	TAG
tRNA^Arg^	1345–1415	71	+	−1		
NAD4L	1415–1712	298	+	−8	ATG	TAA
NAD4	1705–3086	1382	+	−1	ATG	T--
tRNA^His^	3086–3155	70	+	−1		
tRNA^Ser1^	3155–3224	70	+	−1		
tRNA^Leu1^	3224–3297	74	+	−1		
NAD5	3297–5133	1837	+	−5	ATG	TAG
NAD6	5129–5651	523	-	−1	ATG	TAA
tRNA^Glu^	5651–5720	70	-	3		
CYTB	5724–6864	1141	+	1	ATG	TAA
tRNA^Thr^	6866–6940	75	+	1		
tRNA^Pro^	6942–7011	70	-	0		
D-loop	7012–8247	1236				
tRNA^Phe^	8248–8315	68	+	−1		
12S rRNA	8315–9265	951	+	−1	CAC	
tRNA^Val^	9265–9336	72	+	−1		
16S rRNA	9336–11,021	1686	+	0	GTT	
tRNA^Leu2^	11,022–11,097	76	+	−1		
NAD1	11,097–12,069	973	+	1	ATG	TAA
Trna^Ile^	12,071–12,143	73	+	−2		
tRNA^Gln^	12,142–12,213	72	-	−2		
tRNA^Met^	12,212–12,281	70	+	−1		
NAD2	12,281–13,328	1048	+	−3	ATG	TAG
tRNA^Trp^	13,326–13,395	70	+	0		
tRNA^Ala^	13,396–13,465	70	-	0		
tRNA^Asn^	13,466–13,539	74	-	42		
tRNA^Cys^	13,582–13,645	64	-	−1		
tRNA^Tyr^	13,645–13,716	72	-	0		
COX1	13,717–15,301	1585	+	−6	GTG	AGA
tRNA^Ser2^	15,296–15,367	72	-	3		
tRNA^Asp^	15,371–15,441	71	+	1		
COX2	15,443–16,142	700	+	−9	ATG	T--
tRNA^Lys^	16,134–16,209	76	+	0		
ATP8	16,210–16,378	169	+	672	ATG	TAA
ATP6	16,368–16,911	544	+		ATG	TAA

## Data Availability

The original contributions presented in the study are included in the article, mitochondrial genome sequences have been deposited in GenBank under the accession number of PX571992. Further inquiries can be directed to the corresponding author.
